# Global Switch from DICER-dependent MicroRNA to DICER-independent SnoRNA-derived RNA Biogenesis in Malignancy

**DOI:** 10.17912/micropub.biology.000725

**Published:** 2023-02-02

**Authors:** Noel L Godang, Jeffrey D DeMeis, Dominika Houserova, Neil Y Chaudhary, Carly J Salter, Yaguang Xi, Oliver G McDonald, Glen M Borchert

**Affiliations:** 1 Department of Pharmacology, College of Medicine, University of South Alabama, Mobile, AL USA; 2 Department of Genetics, School of Medicine, Louisiana State University Health Sciences Center, New Orleans, LA USA; 3 Stanley S. Scott Cancer Center, Louisiana State University Health Sciences Center, New Orleans, LA USA; 4 Department of Pathology, Sylvester Comprehensive Cancer Center, University of Miami, Miami, FL USA

## Abstract

SnoRNAs are frequently processed into snoRNA-derived RNAs (sdRNAs) that function much like traditional microRNAs (miRNAs). That said, our analyses suggest a global switch from DICER-dependent (predominately miRNA) to DICER-independent (predominately sdRNA) biogenesis/gene regulation in colon cancer. Whereas the expressions of 259 of 288 appreciably expressed miRNAs are significantly decreased (avg. 6.4% of WT) in human colon cancer DICER-KOs, 95 of 103 sdRNAs are conversely, significantly increased (avg. 679.3%) in DICER-KOs as compared to WT. As many diseases are characterized by DICER deficiency, this putative global switch to DICER-independent sdRNA regulations may contribute to an array of human diseases.

**Figure 1. Putative global switch from DICER-dependent miRNA expressions/regulations to DICER-independent sdRNA expressions/regulations when DICER activity is diminished.**
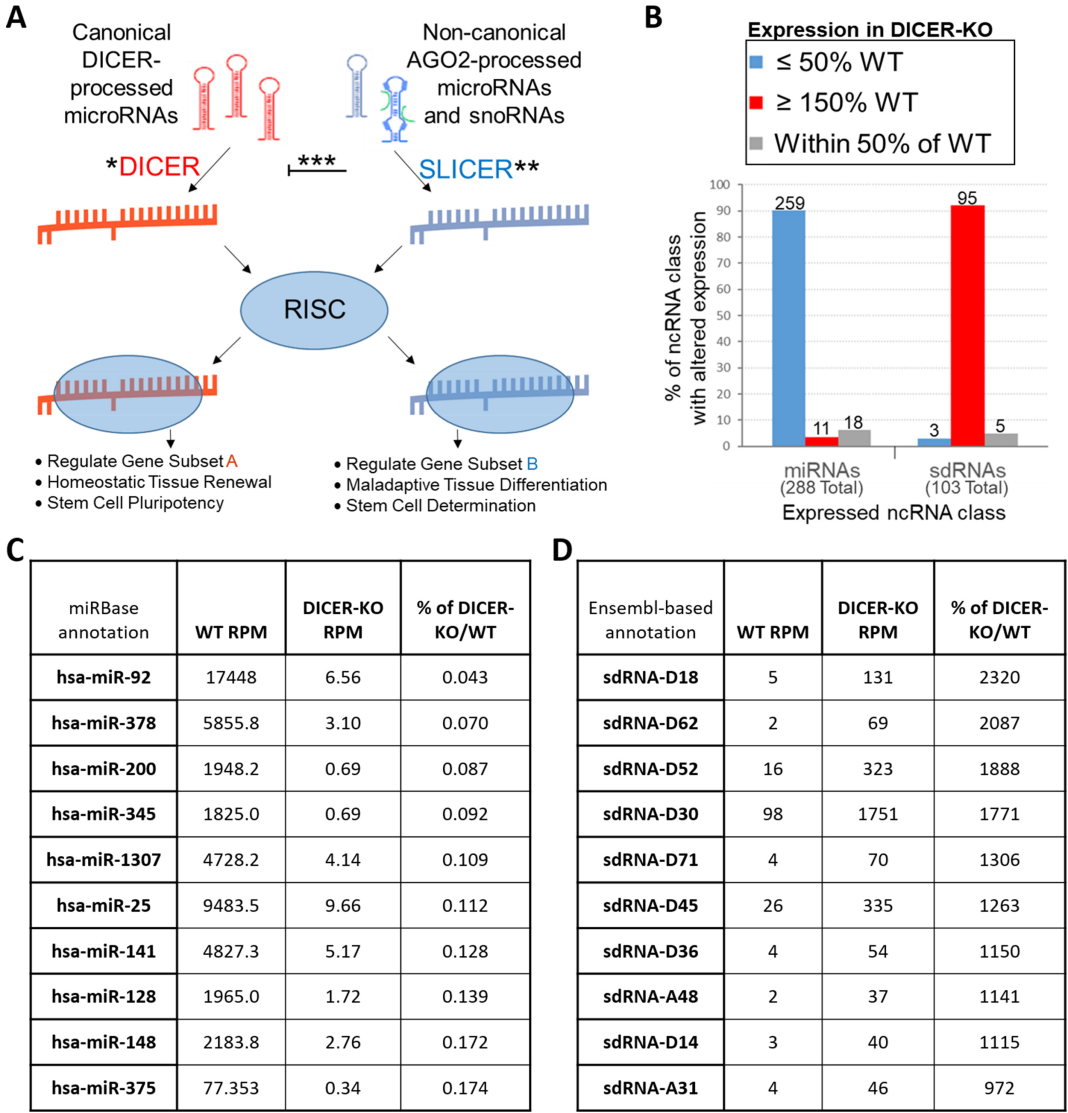
( **A** ) DICER/SLICER RNAi processing switch model. *Typically the predominate form of processing. **SLICER predominates when (i) DICER is inhibited, (ii) DICER is deleted, or (iii) AGO2/SLICER is overexpressed. ***Upregulated AGO2 inhibits DICER activity. ( **B-D** ) WT and DICER-KO human colon cancer cell line (HCT116) small RNA transcriptome comparison. ( **B** ) Graph depicting the percentage of miRNA (left) and sdRNA (right) expressions that are significantly decreased (blue), increased (red), or unchanged (grey) in DICER-KO cells in comparison to WT. The values indicate the number of unique ncRNAs belonging to a category (e.g., 259 of the 288 miRNAs expressed were decreased in DICER-KO cells). ( **C** ) Top 10 downregulated microRNAs in DICER-KO. Annotations taken from miRBase (Release 22.1). ( **D** ) Top 10 upregulated sdRNAs. Annotations taken from Ensembl (Release 108). sdRNA-D18 excised from SNORD18 (ENSG00000200623). sdRNA-D62 excised from SNORD62 (ENSG00000199411). sdRNA-D52 excised from SNORD52 (ENSG00000201754). sdRNA-D30 excised from SNORD30 (ENSG00000277846). sdRNA-D71 excised from SNORD71 (ENSG00000223224). sdRNA-D45 excised from SNORD45 (ENSG00000206620). sdRNA-D36 excised from SNORD36 (ENSG00000200831). sdRNA-A48 excised from SNORA48 (ENSG00000209582). sdRNA-D14 excised from SNORD14 (ENSG00000207118). sdRNA-A31 excised from SNORA31 (ENSG00000199477).

## Description

Mature microRNAs (miRNAs) are single-stranded, ~20 nucleotide (nt) noncoding RNAs (ncRNAs). MiRNAs are primarily excised from longer precursors via DICER to produce mature single-stranded miRNAs that are loaded into the RNA-induced silencing complex (RISC) to direct binding to complementary protein-coding mRNA transcripts for post-translational gene silencing (Borchert et al. 2006; Li and Rana 2014; O’Brien et al. 2018). Notably, several studies have shown that although some individual miRNAs can be upregulated and have an oncogenic function, miRNA expression is frequently globally suppressed in tumor cells compared with normal tissue (Lu et al. 2005; Rupaimoole et al. 2016; Williams et al. 2017). Furthermore, loss of DICER itself is known to contribute to several severe human diseases including multiple malignancies, as well as some autoimmune, neurological, reproductive, cardiovascular, and neoplastic disorders (for example, multiple sclerosis, rheumatoid arthritis, ankylosing spondylitis, Parkinson’s disease, and depression) (Theotoki et al. 2020). Relatedly, global downregulation of DICER-dependent miRNA processing and concurrent reciprocal upregulation of AGO2-dependent, DICER-independent miRNA processing is required for efficient erythropoiesis (Jee et al. 2018; Kretov et al. 2020). Specifically, miR-451 is one of the only a few miRNAs whose maturation is currently known to be wholly independent of DICER (Yang et al. 2010; Zhang et al. 2018; Kretov et al. 2020), and expression of the well-conserved mature miR-451 directly mediates erythroid progenitor differentiation into red blood cells. In short, DICER-independent, AGO2/SLICER processing of the pre-miR-451 hairpin separates it from the biogenesis of standard DICER-dependent miRNAs. The independent regulation of these two separate miRNA-excision pathways allows miR-451 to escape the global downregulation of miRNAs observed during erythropoiesis (Kretov et al. 2020) ( **Figure 1A** ).

Similar in length to miRNA precursors, snoRNAs are an ancient class of 60-300 nt ncRNAs representing one of the most abundant species of small RNA molecules within eukaryotic cells. The canonical function of snoRNAs is to guide homology-directed post-transcriptional editing of ribosomal RNAs (rRNAs) and other ncRNAs in the nucleolus, which ensures accurate translation of proteins by ribosomes. For nearly five decades, it was generally accepted that these housekeeping activities were the primary, if not the sole, function of these molecules (Tollervey and Kiss 1997). Recent discoveries by our lab (Patterson et al. 2017; Coley et al. 2022a) and other groups (Ender et al. 2008; Taft et al. 2009; Brameier et al. 2011; Falaleeva and Stamm 2013; Martens-Uzunova et al. 2013; Martens-Uzunova et al. 2015; Shi et al. 2021), however, indicate that snoRNAs are frequently further processed into shorter ncRNA species ~20 nt in length. These snoRNA-derived RNAs (sdRNAs) likely number in the thousands (Kasukurthi et al. 2019; Kasukurthi et al. 2021; Coley et al. 2022a) and have distinct functions from their snoRNA precursors. Rather than guiding riboside modification of ribosomal and transfer RNAs, sdRNAs appear to guide post-transcriptional silencing of mRNA expression in a homology-driven process that is reminiscent of canonical miRNA-mediated RISC gene silencing (Ender et al. 2008; Taft et al. 2009; Brameier et al. 2011; Falaleeva and Stamm 2013; Martens-Uzunova et al. 2013; Martens-Uzunova et al. 2015; Shi et al. 2021).

In stark contrast to miRNAs, however, recent studies indicate that sdRNAs are often over-expressed in human diseases, including common cancers (recently reviewed in Coley et al. 2022b). As examples, snoRNA sdRNA-93 over-expression contributes to specific breast cancer subtype progressions (Patterson et al. 2017), and similar sdRNA -A24 and -D19b over-expressions have been reported to be associated with enhanced prostate cancer metastasis (Coley et al. 2022a). That said, perhaps the most striking distinction between conventional miRNAs and sdRNAs is that sdRNAs are apparently excised from specific snoRNAs through a DICER-independent process (Taft et al. 2009; Shi et al. 2021).

The data presented here strongly agree with previous reports (Taft et al. 2009; Shi et al. 2021) suggesting that sdRNAs are excised from snoRNAs by a DICER-independent, AGO2/SLICER-driven pathway clearly distinct from the typical DICER-dependent biogenesis of miRNAs. Independent analysis of the small RNA transcriptomes of human colon cancer HCT116 WT and DICER-KO cells identified 288 miRNAs and 103 sdRNAs to be appreciably expressed (≥ 30 RPM) in WT and/or KOs. Notably, whereas we find the expressions of 259 of 288 miRNAs to be significantly decreased (avg. 6.4% of WT) in HCT116 DICER-KOs, we find 95 of 103 sdRNA expressions to be conversely, significantly increased (avg. 679.3%) in DICER-KO HCT116 cells as compared to WT ( **Figure 1B-D** ).

While our group’s initial work on sdRNAs focused primarily on establishing their relevance in malignancy (Patterson et al. 2017; Coley et al. 2022a, Coley et al. 2022b), we have recently reassessed how (and if) sdRNAs differ from miRNAs electing to take a much broader look at sdRNAs and miRNAs in health and disease. That said, we were stunned to find (in the analyses presented here) that the near total loss of miRNA expression observed during DICER impairment is accompanied by a reciprocal, global increase in sdRNA expressions. While our and others’ previously published sdRNA analyses strongly implicate a RNAi-based mechanism for sdRNA that is reminiscent of miRNAs (Patterson et al. 2017; Coley et al. 2022a; Ender et al. 2008; Taft et al. 2009; Brameier et al. 2011; Falaleeva and Stamm 2013; Martens-Uzunova et al. 2013; Martens-Uzunova et al. 2015; Shi et al. 2021), the data presented here indicate that unlike miRNAs, many sdRNAs are excised from snoRNAs by a DICER-independent mechanism. Importantly, DICER deficiency and/or global reductions in miRNAs occur across many human diseases and our current findings strongly suggest a fundamental switch from DICER-dependent miRNA regulation of gene expression to AGO2-dependent sdRNA regulation when DICER activity is diminished. Our data now raise the possibility of a global switch from DICER-dependent miRNA regulations associated with normal cellular metabolism to DICER-independent sdRNA regulations triggered during malignant transformation when DICER activity is diminished (e.g., sdRNA-A24 repression of CDK12 a cell cycle regulator and known tumor suppressor which plays a role in genomic stability and is mutated in ~6% of patients with metastatic castrate-resistant PCa (Coley et al. 2022a)).

## Methods

A single FASTA file comprising all annotated human miRNAs contained within the miRNA registry (Kozomara and Griffiths-Jones 2011) and all human snoRNAs currently annotated in Ensembl (Cunningham et al. 2015) was assembled. Alignments between snoRNAs and miRNAs and individual small RNA-seq reads were performed on the Alabama Supercomputer Center SGI UV 2000 and DMC cluster and obtained via Basic Local Alignment Search Tool (BLAST+) using the following parameters: 100% identity, word_size = 6, ungapped, and evalue = 0.001 (Camacho et al. 2009). All accepted BLAST+ alignments were restricted to perfect matches (100% identity) between 16 and 32 nts. The frequency of alignments to putative sdRNA loci across each full-length snoRNA was calculated by counting reads defined as ≥16 nts and perfect matches (100% identity) and sdRNAs called as previously defined (Patterson et al. 2017). Publicly available next-generation small RNA deep-sequencing libraries were obtained from the NCBI Sequence Read Archive (SRA) (www.ncbi.nlm.nih.gov/sra/). These included HCT116 WT (SRR3174964) and DICER-KO (SRR3174968) small RNA transcriptomes. Individual miRNAs and sdRNAs not expressed at ≥30 RPM in either library were excluded. BLAST-based alignments were confirmed by independently employing SURFR (Kasukurthi et al. 2019; 2021). The Short Uncharacterized RNA Fragment Recognition (SURFR) tool comprehensively profiles ncRNA-derived RNAs from input RNA-seq data. SURFR analysis of HCT116 WT (SRR3174964) and DICER-KO (SRR3174968) small RNA transcriptomes returned expression in reads per million (RPM) for each sdRNA and miRNA detected with 100% of miRNA and sdRNA expressions agreeing within 3.1% of BLAST alignment-based values. RStudio was used to calculate differential expression of BLAST alignment-based values. Returned results strictly required sdRNAs and miRNAs to be expressed at ≥ 30 RPM in at least one library and all sdRNAs and miRNAs were classified as (1) upregulated; ≥ 150% expression in DICER-KO as compared to WT, (2) downregulated; ≤ 50% expression in DICER-KO as compared to WT, or (3) unchanged; expression in DICER-KO within 50% of WT.
